# Intraoperative bispectral index monitoring and time to extubation after cardiac surgery: secondary analysis of a randomized controlled trial

**DOI:** 10.1186/1471-2253-14-79

**Published:** 2014-09-18

**Authors:** Jennifer L Vance, Amy M Shanks, Derek T Woodrum

**Affiliations:** 1 Department of Anesthesiology, University of Michigan Medical School, 1H247 UH Box 5048, 1500 East Medical Center Drive, Ann Arbor, MI 48109, USA

**Keywords:** BIS, Cardiac surgery, Fast-track extubation

## Abstract

**Background:**

Fast track recovery is a care process goal after cardiac surgery. Intraoperative anesthetic depth may impact recovery, but the impact of brain monitoring on time to extubation and intensive care unit (ICU) length of stay after cardiac surgery has not been extensively studied. Our goal was to determine if BIS-guided anesthesia improves time to extubation compared to MAC-guided anesthesia in a cardiac surgery population.

**Methods:**

In this secondary outcome analysis of a randomized controlled study, we analyzed 294 patients undergoing elective coronary bypass grafting, valve replacements, and bypass plus valve replacements at a single tertiary referral center between February 1, 2009 and April 30, 2010. We analyzed cardiac surgery patients that had been randomized to BIS-guided anesthesia alerts (n = 131) or MAC-guided anesthesia alerts (n = 163). The primary outcome measure was time to extubation in the BIS-guided and anesthetic concentration-guided groups. Secondary outcomes were length of stay in the ICU and total postoperative hospital length of stay.

**Results:**

Valid extubation time data were available for 247 of 294 patients. The median [IQR] time to extubation was 307 [215 to 771] minutes in the BIS group and 323 [196 to 730] minutes in the anesthetic concentration group (p = 0.61). The median [IQR] ICU length of stay was 54 [29 to 97] hours versus 70 [44 to 99] hours (p = 0.11). In terms of postoperative hospital length of stay, there was no difference between the groups with median [IQR] times of 6 [5-8] days (p = 0.69) in each group.

**Conclusions:**

The use of intraoperative BIS monitoring during cardiac surgery did not change time to extubation, ICU length of stay or hospital length of stay. Data regarding BIS monitoring and recovery in an exclusively cardiac surgery population are consistent with recent effectiveness studies in the general surgical population.

**Trial registration:**

ClinicalTrials.gov number NCT00689091.

## Background

The past two decades have seen an increasing interest in early endotracheal extubation after cardiac surgery, typically defined as extubation within 8 hours of the end of surgery. Early extubation is associated with decreased intensive care unit (ICU) length of stay (LOS), hospital LOS, and thus the potential for improved recovery and decreased resource utilization [1,2]. Risk factors for prolonged intubation include need for inotropic support, excessive bleeding, and atrial arrhythmias; these factors can thus help predict suitability for early extubation [3]. Early extubation has been shown to be safe compared to standard post-cardiac surgery extubation [2,4,5]. While intraoperative anesthetic techniques have evolved to emphasize fast track recovery [6], the role of brain monitoring in extubation and length of stays after cardiac surgery has not been well studied. In the B-Aware [6], B-Unaware [7], and BAG-RECALL [8] trials, the use of BIS monitoring was not associated with a reduction in the amount of anesthesia administered or a decreased hospital length of stay, but only some of the patients in these trials underwent cardiac procedures.

The Bispectral Index (BIS) monitor (Covidien, Boulder, CO, USA) has been used as a surrogate for depth of anesthesia in and has also been posited to improve recovery from surgery and general anesthesia. The BIS monitor could have direct or indirect effects on recovery. In terms of direct effects, initial efficacy studies have demonstrated that BIS monitoring can reduce anesthetic use and, consequently, the time to extubation and other recovery parameters in noncardiac surgery [9,10]. In other words, consciousness recovers faster by using less anesthetic. However, BIS monitoring could also have indirect effects on recovery across different surgical populations. Such effects could be mediated through cognitive phenotypes that might play a more important role in determining extubation in an ICU setting. For example, a patient with delirium in the ICU may be less likely to be extubated. Thus, if the intraoperative use of the BIS monitor affects intermediate cognitive phenotypes such as postoperative delirium, it could alter the time to extubation in the postoperative period of critical care. Indeed, a recent study reported on the protective odds ratio conferred by BIS-guided anesthesia when studying postoperative delirium in cardiac surgical patients [11]. In a recent study of noncardiac patients, it was shown that BIS-guided anesthesia was associated with a reduction in the concentration of anesthetics delivered, with improvements in cognition at three months after surgery [12]. Thus, the intraoperative use of the BIS monitor has been linked to a number of outcomes that extend far into the postoperative period. As such, we hypothesized that the use of the BIS monitor during cardiac surgery could also have relevance to the postoperative period in terms of reducing recovery times. The primary aim of this study was to determine whether the intraoperative use of the BIS improved time to extubation after cardiac surgery compared to an anesthetic concentration protocol. Secondary outcomes included differences in length of stay in the intensive care unit and total postoperative length of stay in the hospital.

## Methods

### Study design and patient cohort

The Michigan Awareness Control Study (MACS; ClinicalTrials.gov Trial Registration Number NCT00689091) was a randomized controlled effectiveness trial comparing alerting protocols based on the BIS (trigger for BIS > 60) and anesthetic concentrations (minimum alveolar concentration [MAC] <0.5). The trial included adults undergoing general anesthesia for an unselected surgical population. The major conclusion of MACS was that there was no detectable difference in the incidence of definite awareness or recovery variables between monitoring protocols based on either BIS values or anesthetic concentration [13]. The current study is a secondary analysis of cardiac patients from this cohort and ethical approval (HUM00044647) was provided by the Institutional Review Board of the University of Michigan, Ann Arbor, USA (Chairperson Michael Geisser) on 26 September 2011. Patient consent was waived as this was a secondary analysis of de-identified data. Eligible patients included all elective coronary bypass grafting (CABG), valve replacements, and CABG plus valve replacements that were part of the primary study lasting from February 1, 2009 until April 30, 2010. Time during recruitment differs from the parent study because recruitment of patients at the Cardiovascular Center of the University of Michigan Health System began later than other study sites. Exclusion criteria included emergency cases, aortic dissections, or hypothermic circulatory arrest cases, since these cases are typically not candidates for early extubation.

Perioperative data were collected using Centricity® (General Electric Healthcare, Waukesha, WI, USA), an anesthesia information management system. All information is entered by anesthesia personnel (residents, fellows, nurse anesthetists and attendings). For each data point, the user may select from a predefined list or enter free-text information to describe any clinical variable. A history and physical examination is electronically documented for every case. Additional information was obtained from the Society of Thoracic Surgeons (STS) National Cardiac Database. The database collects more than 350 variables from approximately 95 percent of all adult cardiac surgery centers throughout the United States. Each site voluntarily enters patient data using pre-specified definitions and certified software systems; the data are sent semiannually to the STS Data Warehouse and Analysis Center at the Duke Clinical Research Institute. The data subsequently undergo a series of quality checks before being included in the national sample.

### Surgical procedures

Patients underwent surgery either via a median sternotomy or via a thoracotomy (for redo mitral valve operations or patients’ cosmetic-driven request). All cases were performed using cardiopulmonary bypass (CPB), with antegrade and/or retrograde cardioplegia for myocardial protection. CPB utilized nonpulsatile flow for all cases. Heparin was administered to achieve and maintain an activated clotting time >450s. Both nasal and bladder temperature probes were placed for monitoring, and the patient’s temperature was reduced to between 32-35°C during CPB. After termination of CPB, protamine 1–1.3 mg/100 units heparin was administered. All patients were transported to the ICU intubated once hemodynamically stable.

### Anesthetic technique and interventions

Induction of anesthesia was performed with midazolam, fentanyl, pancuronium, with or without propofol or etomidate, titrated to loss of consciousness and muscle relaxation. Anesthesia was subsequently maintained with isoflurane, and additional fentanyl and pancuronium as needed. Isoflurane was continued during cardiopulmonary bypass. All patients were sedated with propofol infusions at varying rates at the end of the case; sedation was continued in the ICU until the patients were weaned for tracheal extubation.

In the Michigan Awareness Control Study, patients were randomized to a BIS-guided group or MAC-guided group. Automated pages were sent to the intraoperative anesthesia provider when the median BIS rose above 60 for a 5-minute epoch or when the effective age-adjusted MAC fell below 0.5 (in the MAC-guided group). There was no lower value alert for the BIS group and no upper value alert for the MAC-guided group.

### Outcomes

The primary outcome measure for the current study was the time to extubation across the two groups (BIS-guided, MAC-guided). Secondary outcomes included length of stay in the ICU and total postoperative length of stay in the hospital. We also compared the groups in terms of factors that could impede early extubation, including vasopressor usage at the end of the case and excessive bleeding, for which we used intraoperative blood product usage as a surrogate. We defined high blood product utilization as greater than 3 units of fresh frozen plasma (FFP) or greater than 4 units of packed red blood cells (pRBCs). We arrived at these values based on previous studies of average blood utilization in cardiac surgery [14-16].

### Statistical analysis

Statistical analysis was performed using SPSS statistics version 19 (IBM Corp, Somers, NY, USA). Data from the anesthesia information system and the STS database from our institution were merged together for all patients that met the inclusion criteria using a third-party honest broker system. After the merge was completed, all identifiers were removed prior to analysis by the study team. Patients without valid data in both the anesthesia information system and the STS database were excluded from this analysis. The Kolmogorov-Smirnov statistic was used to determine normality for all continuous data elements. If the p-value was significant (<0.05), the assumption of normality was violated and a non-parametric test (Mann Whitney U test) was used. For categorical variables, a two-tailed Pearson chi-square or Fisher’s Exact test were used as appropriate. A p-value of <0.05 was considered statistically significant.

## Results

Data were captured for a total of 294 patients divided into two groups: those with MAC-based alerts (163 patients) and those with BIS-based alerts (131 patients). The two groups were well matched with respect to age, sex, body mass index, smoking history, airway characteristics, as well as the number and severity of comorbidities such as cardiac, pulmonary, and renal disease (Table 1).

**Table 1:** Patient characteristics

	**MAC (N = 163)**	**BIS (N = 131)**	**P Value**	**Odds ratio**	**95% CI**
Male	102 (62.6%)	92 (70.2%)	0.169	1.41	0.86 to 2.30
Body mass index (median)	28.7	28.0	0.411		
Age (median)	63.0	64.0	0.824		
Preoperative β-blockers	99 (60.7%)	93 (71.0%)	0.66	1.58	0.97 to 1.88
Current smoker	11 (6.7%)	10 (7.6%)	0.77	1.14	0.47 to 2.78
Former smoker	68 (41.7%)	64 (48.9%)	0.221	1.34	0.84 to 2.12
Chronic pain	17 (10.4%)	7 (5.3%)	0.113	0.49	0.20 to 1.21
Stroke/transient ischemic attack	11 (6.7%)	8 (6.1%)	0.824	0.90	0.35 to 2.30
Diabetes mellitus	48 (29.4%)	30 (22.9%)	0.206	0.71	0.42 to 1.21
Renal failure	25 (15.3%)	33 (22.9%)	0.098	1.64	0.91 to 2.96
Asthma	10 (6.3%)	8 (6.2%)	0.986	0.99	0.38 to 2.60
**Sleep apnea**	50 (30.9%)	34 (26.2%)	0.377	0.79	0.48 to 1.33
w/ CPAP	16 (9.8%)	14 (10.7%)	0.806	1.10	0.52 to 2.34
**Lung disease**	109 (66.9%)	89 (67.9%)	0.846	1.05	0.64 to 1.72
Dyspnea	101 (62.0%)	83 (63.4%)	0.806	1.06	0.66 to 1.71
Pulmonary hypertension	16 (9.8%)	14 (10.7%)	0.806	1.10	0.52 to 2.34
Chronic obstructive pulmonary disease	14 (8.4%)	13 (9.2%)	0.971		
Other lung disease	7 (4.3%)	11 (8.4%)	0.145	2.04	0.77 to 5.43
**Cardiovascular disease**	153 (93.9%)	125 (95.4%)	0.559	1.36	0.48 to 3.85
Valvular heart disease	94 (57.7%)	73 (55.7%)	0.738	0.92	0.58 to 1.47
Coronary artery disease	80 (49.1%)	73 (55.7%)	0.257	1.31	0.82 to 2.07
Congestive heart failure	42 (25.8%)	32 (24.4%)	0.793	0.93	0.55 to 1.58
Dysrhythmia	37 (22.7%)	31 (23.7%)	0.845	1.06	0.61 to 1.82
Angina	35 (21.5%)	33 (25.2%)	0.452	1.23	0.71 to 2.12
Myocardial infarction	30 (18.4%)	28 (21.4%)	0.525	1.21	0.68 to 2.14
Orthopnea	14 (8.6%)	5 (3.8%)	0.098	0.42	0.15 to 1.21
Peripheral vascular disease	6 (3.7%)	9 (6.9%)	0.217	1.93	0.67 to 5.57
Endocarditis	6 (3.7%)	6 (4.6%)	0.699	1.26	0.40 to 3.99
**Ejection fraction**					
<30	11 (6.7%)	10 (7.8%)	0.947^*^		
30-50	37 (22.7%)	29 (22.5%)			
>50	115 (70.6%)	90 (69.8%)			
**Airway**					
Previous difficult intubation	0	0	0		
Limited mouth opening	3 (1.8%)	5 (3.8%)	0.301	2.12	0.50 to 9.02
Limited thyromental distance	12 (7.4%)	8 (6.1%)	0.671	0.82	0.32 to 2.07
Limited neck flexion	1 (0.6%)	2 (1.4%)	0.627		
Limited neck extension	16 (9.6%)	14 (9.9%)	0.627		
Limited jaw protrusion	18 (11.0%)	11 (8.4%)	0.335		
Intraoperative view grade 3 or 4	8 (5.0%)	3 (2.4%)	0.254		
Intraoperative mask grade 3 or 4	6 (3.7%)	5 (3.8%)	0.960		

*MAC* minimum alveolar concentration, *BIS* Bispectral index, *CI* confidence intervals.

*Ejection Fraction is an ordinal variable with three categories as listed above. There was no significant difference between the MAC and BIS patients across the three ordinal categories.

In terms of time to extubation, the primary outcome, there was no statistically significant difference between the two groups (Table 2). Of the 294 patients, valid extubation time data were available for 247 patients. The median [inter quartile range (IQR)] times to extubation in the BIS-based alert and MAC-based alert groups were 307 [215 to 771] and 323 [196 to 730] minutes, respectively; p = 0.610 (Table 2). There was also no statistically significant difference in the secondary outcome of ICU length of stay. The median [IQR] length of stay was 54 [29 to 97] hours versus 70 [44 to 99] hours; p = 0.108 (Table 2). Finally, there was no difference between the groups for the secondary outcome of total postoperative hospital length of stay with median [IQR] times of 6 [5-8] days in each group; p = 0.688 (Table 2).

**Table 2:** Recovery parameters

	**Bispectral index**	**Minimum alveolar concentration**	**p value**
Time to extubation (min)	307 [215 to 771]	323 [196 to 730]	0.610
Intensive care unit length of stay (hrs)	54 [29 to 97]	70 [44 to 99]	0.108
Hospital length of stay (days)	6.0 [5.0-8.0]	6.0 [5.0-8.0]	0.688

There are many factors that can prolong postoperative intubation following cardiac procedures, including: presence of an intra-aortic balloon pump (IABP), excessive bleeding, vasopressor use, pre-existing lung disease, prolonged surgical time, or excessive depth of anesthesia [4]. The two groups were well matched in key areas. Use of an intra-aortic balloon pump was an exclusion criterion. There were no significant differences between the groups in terms of lung disease (Table 1). The two groups had no significant differences in either the FFP or pRBC use categories (Table 3). There was also no difference in total operating room time (as measured by total anesthesia time) [Table 4], or vasopressor use at the end of the case (as measured by defined dosages prior to transport) [Table 5]. In terms of any effect of anesthetic depth on time to extubation, the median age-adjusted MAC was not significantly different between the BIS-based alert and MAC-based alert groups: 0.45 [0.37 to 0.56] versus 0.46 [0.37 to 0.56]; p = 0.738. The median BIS values between the BIS-alert and MAC-alert groups were 40 [37–42] versus 42 [38–45]; p = 0.026. This statistically significant difference is highly unlikely to be of clinical significance, since the typical range of BIS values consistent with general anesthesia is 40–60; note also that the BIS values were not visible to the MAC allocation group.

**Table 3:** Intraoperative high blood product utilization

	**BIS**	**MAC**	**p value**	**Odds ratio**	**95% CI**
High blood product utilization	25 (19.1%)	28 (17.2%)	0.673	1.14	0.63 to 2.10
Intraoperative FFP >3 units	13 (9.9%)	10 (6.1%)	0.229	1.69	0.71 to 3.98
Intraoperative pRBCs >4 units	22 (16.8%)	27 (16.6%)	0.958	1.02	0.55 to 1.88

High Blood Product Utilization defined as: >3 units fresh frozen plasma (FFP) or >4 units packed red blood cells (pRBCs) intraoperatively.

**Table 4:** Total anesthesia time

	**Bispectral index**	**Minimum alveolar concentration**	**P value**
Total time under anesthesia (minutes)	363 [304 to 445]	346 [290 to 413]	0.165

**Table 5:** High vasopressor requirements

	**BIS**	**MAC**	**p value**	**Odds ratio**	**95% CI**
High vasopressor requirements (number of patients and % of total)	33 (25.2%)	35 (21.5%)	0.452	1.23	0.72 to 2.12

High vasopressor requirement defined as the dose of vasopressor needed within 15 minutes of transport to the intensive care unit (norepinephrine >0.05 mcg/kg/min, phenylephrine >40 mcg/min, vasopressin >2 units/min, epinephrine >0.02 mcg/kg/min).

## Discussion

This study did not identify a statistically significant difference in time to extubation after cardiac surgery between BIS-guided and MAC-guided groups, which were well matched in terms of comorbidities. There were also no statistically significant differences in ICU or total postoperative hospital lengths of stay.

In non-cardiac surgery patients, initial efficacy studies suggested that BIS-monitoring may decrease time to extubation [9,10]. However, subsequent analysis [17] of large effectiveness studies did not show that BIS confers a benefit in overall time to recovery. Time to extubation following *cardiac* surgery has been an area of research in many studies, but few involve brain monitoring. Ender, et al. [4] have described how the implementation of goal-focused processes (creation of a specific cardiac-recovery post-anesthesia care unit (PACU) and modifying intraoperative anesthesia) can greatly decrease time to extubation in selected patients (stable hemodynamics without inotropic support, no excessive bleeding, and normothermia). Gooi et al. [18] showed that the preoperative identification of a lower-risk patient cohort allowed for earlier extubation in that fast-track group. However, neither study utilized brain monitoring.

Wong, et al. [3] showed that increased age, female sex, use of inotropes, excessive bleeding, atrial arrhythmias, and presence of an intra-aortic balloon pump were associated with increased times to extubation. These variables represent potential confounders, but the groups in our study showed no difference in age, sex, vasopressor use, excessive bleeding, or dysrhythmias (Table 1). Also, the groups were similar in the median age-adjusted MAC and total anesthesia time (Table 4) showing that the anesthetic depth or duration had no effect on extubation times.

Although most time-to-extubation studies in cardiac surgery patients have not included brain monitoring, a secondary analysis by Villafranca, et al. from the BAG-RECALL trial demonstrated that the use of a BIS protocol during cardiac surgery in a Canadian hospital did not improve extubation time compared to a protocol based on age-adjusted 0.7 MAC [19]. A power analysis showed that 50 patients in each group would be required to detect a 30 minute difference in extubation time at a power of 0.95. Both their study and the present investigation greatly exceed 50 patients in each cohort. The current study confirms the previous findings and adds to the literature by demonstrating comparable results in a separate population with a protocol based on an even lower MAC threshold (age-adjusted 0.5 MAC). Thus, two independently conducted analyses of larger randomized controlled trials suggest no clear benefit to intraoperative BIS monitoring during cardiac surgery relative to the outcome of early extubation. Our study additionally demonstrates no difference in the secondary outcomes of ICU length of stay or overall hospital stay. As BIS is not routinely employed in our ICU, it cannot be concluded from the current study whether BIS monitoring in the ICU is beneficial for earlier extubation time.

There are limitations to this study. First, as noted, the BIS monitor was not continued in the ICU to monitor depth of sedation postoperatively. Furthermore, at our institution, there is no set dose and no strict protocol that dictates depth of sedation. However, we note that a study in which ICU propofol doses were not different between groups, the use of a BIS monitor was not associated with shorter time to extubation [20]. Second, patients who arrive late in the evening are often sedated and intubated overnight because of staffing issues when they may have otherwise been extubated. Thus, time to extubation or ICU length of stay are potentially skewed by these confounds. In a previous study, it was shown that patients who were admitted to the ICU in the evening tended to be intubated longer independent of comorbidities and surgical factors [18,19]. Third, this was a secondary outcome analysis of a prospective trial with a primary outcome of intraoperative awareness with explicit recall; data regarding recovery and length of stay must therefore be interpreted cautiously. However, our approach of secondary analysis eliminates any practitioner bias of trying to achieve the outcome of early extubation. Fourth, our anesthetic practice in cardiac surgery does not routinely involve total intravenous anesthesia; results may be different in this population. Finally, because the initial trial involved awareness prevention, there were no lower limit alarms for BIS and upper limit alarms for MAC, which might have altered anesthetic management in a way that influenced time to extubation. However, a similar secondary analysis of a BIS vs. MAC alarm trial that incorporated *both* upper and lower BIS limits similarly failed to find an association between BIS utilization and time to extubation after cardiac surgery [19].

## Conclusion

There was no difference in time to extubation between BIS-guided and MAC-guided anesthetics in a patient population well matched for comorbidities and for potential confounders for prolonged intubation. There also were no statistically significant differences in ICU and total post-operative hospital length of stay. Data from the current study are impactful because they suggest that the use of the BIS monitor during cardiac surgery does not confer an advantage over anesthetic-concentration guided protocols in earlier extubation times.

## Competing interests

The authors declare that they have no competing interests.

## Authors’ contributions

JV conceived the study, participated in its design and helped to draft the document. AS performed the statistical analyses and helped with results analysis. DW participated in results analysis and helped to draft the document. All authors read and approved the final manuscript.

## Pre-publication history

The pre-publication history for this paper can be accessed here:

http://www.biomedcentral.com/1471-2253/14/79/prepub
